# Clozapine rebound catatonia or malignant neuroleptic syndrome? Case report

**DOI:** 10.1192/j.eurpsy.2026.12132

**Published:** 2026-07-31

**Authors:** R. Softic

**Affiliations:** Psychiatry, University Clinical Center Tuzla, Tuzla, Bosnia and Herzegovina

## Abstract

**Introduction:**

Abrupt discontinuation of clozapine is not recommended except in cases of serious, life threatening side effects. Catatonia and malignant neuroleptic syndrome (MNS) are severe neuropsychiatric conditions that can present with overlapping clinical features, including stupor, mutism, rigidity, and altered consciousness. However, their etiologies and therapeutic implications differ substantially. While MNS is typically triggered by potent dopamine D2 receptor antagonism and requires discontinuation of neuroleptic therapy, catatonia may arise in the context of primary psychiatric disorders or as a rebound phenomenon following abrupt clozapine withdrawal. Distinguishing between these syndromes is critical to guide treatment.

**Objectives:**

The aim is to present the case of a patient with schizophrenia who developed overlapping symptoms of catatonia and neuroleptic malignant syndrome.

Case presentation

We describe a male patient, 45 years of age, with chronic schizophrenia who had been maintained on clozapine therapy up to 450 mg/day for over 18 years. After psycho-social stress and certain worsening of his mental condition clozapine was abruptly discontinued and fluphenazine 7.5 mg/day was introduced into the therapy. He became less alert and nonresponsive. A worsening of negative symptoms of schizophrenia was suspected and he was hospitalized in the psychiatric ward of a general hospital. On the third day of hospitalization, he begins to have difficulty breathing. CT angiography indicated a pulmonary embolism. The patient is out of contact, sweating, febrile up, and impaired in consciousness. He was then referred to the university clinic for neurology. Due to difficulty breathing, he was immidiately transferred to the ICU. Psychiatrist was consulted. Mental state examination: The patient is mechanically restrained. Not responsive. He actively resists when we try to open his eyes. He is febrile at 38.2 ^o^C. After the restraints are removed, the following was noted: a marked rigor of a plastic type (flexibilitas cerea) exists in all muscles.The “psychological pillow” phenomenon is present.

Treatment: After the administration of diazepam i.v. 2x 10 mg, muscle rigidity partially subsided. It was decided to reintroduce clozapine, and the dose was gradually increased. The patient’s condition improved daily, and was transferred to the university psychiatric clinic for additional stabilization. He was discharged in good general condition.

**Conclusions:**

This case highlights the diagnostic challenge of differentiating clozapine-sensitive catatonia from MNS. Short-lived fever, partial benzodiazepine responsiveness, and clinical recovery following clozapine reintroduction favored a diagnosis of rebound catatonia rather than MNS. Awareness of this phenomenon is essential to avoid misdiagnosis and inappropriate treatment.

**Disclosure of Interest:**

None Declared

